# Characterization of Plant-Based Nutritional Bar Formulated with Chickpea and *Justicia spicigera* Powder

**DOI:** 10.3390/foods14244177

**Published:** 2025-12-05

**Authors:** Minerva Bautista-Villarreal, Juan G. Báez-González, Judíth Miguel Cerezo, Sergio A. Galindo-Rodríguez, Andrés M. Piña-Barrera

**Affiliations:** 1 Departamento de Alimentos, Facultad de Ciencias Biológicas, Universidad Autónoma de Nuevo León, Av. Pedro de Alba s/n, Cd. Universitaria, San Nicolás de los Garza 66455, Mexico; minerva.bautistavl@uanl.edu.mx (M.B.-V.); juan.baezgn@uanl.edu.mx (J.G.B.-G.); 2 Departamento de Nutrición y Estilo de Vida, Facultad de Ciencias de la Salud, Universidad de Montemorelos, Av. Libertad 1300, Montemorelos 67515, Mexico; 1200203@alumno.um.edu.mx; 3 Laboratorio de Nanotecnología, Facultad de Ciencias Biológicas, Universidad Autónoma de Nuevo León, Av. Pedro de Alba s/n, Cd. Universitaria, San Nicolás de los Garza 66455, Mexico; sagrod@yahoo.com.mx

**Keywords:** functional snack, legume-based product, antioxidant activity, functional food, vegan

## Abstract

The growing interest in functional foods has led to the development of plant-based products aimed at improving nutritional status and helping to prevent chronic diseases. This study aimed to develop a vegan nutritional bar (VNB) formulated with chickpea (*Cicer arietinum*) and *Justicia spicigera* powder (JP) (VNB_3.2) and evaluate its proximate composition (AOAC methods), water activity, texture profile, color, and percentage of radical scavenging activity as antioxidant capacity (DPPH and ABTS methods). The formulation was designed to enhance the nutritional and functional properties of the bar by incorporating legume-based protein and natural antioxidants. The VNB_3.2 formulation (with 3.2% *w*/*w* JP) demonstrated a significant increase in antioxidant capacity (77.48 ± 6.86% inhibition) compared to the control (47.61 ± 1.13%). Proximate analysis showed higher protein content (14.31 ± 0.01%) and fat (26.39 ± 0.33%) in VNB_3.2, with a slightly lower carbohydrate (41.13 ± 0.35%) content and crude fiber (3.51 ± 0.121%). Water activity remained below the microbial safety threshold in both samples (*a_w_* < 0.76), with VNB_3.2 exhibiting better stability. Color parameters were markedly modified by *Justicia spicigera*, resulting in a darker appearance and lower a* and b* values. Texture analysis showed acceptable hardness and deformation, supporting the physical stability of the product. The results demonstrated that the inclusion of *Justicia spicigera* contributed significantly to antioxidant activity without compromising textural or physicochemical properties. This study supports the development of plant-based functional foods with improved health-promoting properties.

## 1. Introduction

The consumption of functional foods (FFs) has significantly increased in recent years, mainly due to the growing prevalence of non-communicable chronic diseases and the persistence of malnutrition problems in various regions of the world [1]. FFs are defined as foods that provide additional health benefits beyond basic nutritional value and have gained considerable relevance due to their potential to prevent diseases and improve overall well-being [2]. These types of foods, which contain natural bioactive compounds such as polyphenols, fiber, or antioxidants, to name a few, represent a viable and accessible strategy to complement nutrient deficient diets and promote public health [3]. Plant-based ingredients have received special attention within the FFs domain, particularly due to their phytochemical content (i.e., secondary metabolites) and low environmental impact, among other factors. In this context, the development of food products that are plant-based and natural bioactive compounds offers a promising solution to improve quality of life, especially in populations with limited access to high-quality nutritious foods [4]. Malnutrition—in the context of the deficiency in essential macro- and micronutrient intake—remains one of the leading causes of disease and developmental delays in many countries. One of these diseases is anemia, which remains a significant public health problem worldwide, affecting approximately one in four people, with women of reproductive age and young children being the most vulnerable groups [5]. In Mexico, recent national surveys report an anemia prevalence of 15.8% among adult women and 6.8% among preschool children [6], highlighting the need for accessible, nutrient-rich foods to address the nutritional deficiencies associated with this disease.

FFs such as plant-based nutritional bars (NB) represent an effective strategy to address these deficiencies by offering accessible, shelf-stable, and culturally acceptable food products. In this context, this market is projected to reach US $20.05 billion by 2030, with a compound annual growth rate (CAGR) of 5.9% between 2025 and 2030 positioning it as one of the most important snack products [7,8]. The NB is among the most convenient delivery systems for functional bioactive compounds, offering stability, ease of consumption, and portability. Their formulation aims to balance essential macronutrients (proteins, carbohydrates, and lipids) while incorporating functional ingredients that provide additional health benefits [9]. In this regard, chickpea (*Cicer arietinum*) is an ideal ingredient for such formulations due to its high protein content, balanced amino acid profile, fiber content, and the presence of bioactive compounds such as polyphenols and saponins [9,10,11]. Recent studies have shown that its inclusion not only improves the nutritional quality of food products but also enhances sensory acceptability and antioxidant stability. As a base ingredient, chickpeas provide protein, complex carbohydrates, minerals, and bioactive compounds that support general nutritional status [12,13].

On the other hand, the incorporation of *Justicia spicigera* can enrich the product by including biologically active compounds with antioxidant capacity such as kaempferol glycosides and anthocyanins such as cyanidin 3-O-glucoside and cyanidin 3,5-di glucoside present in the plant, improving the body’s ability to prevent and combat chronic diseases from early stages [14]. *Justicia spicigera*, commonly known as muicle, is a native plant of Mesoamerica traditionally used for medicinal purposes to treat gastrointestinal, respiratory, and circulatory conditions, especially used in the treatment of anemia [15,16,17]. Although research has addressed its phytochemical composition and biological activities, there is still limited information regarding its complete nutritional profile, especially regarding the concentration of carbohydrates, proteins, and minerals. However, recent studies have confirmed that its leaves and extracts contain phenolic compounds and flavonoids with high antioxidant capacity, anti-inflammatory activity, hepatoprotective, and antilipidemic effects [18]. Although its toxicity level has not been clearly reported, extracts of *Justicia spicigera* have been used in food. For example, Castro-Alatorre et al. (2021) [14] successfully applied microencapsulated ethanolic extract in yogurt and gelatin, obtaining an acceptable sensory result. Bernardo-Mazariegos et al. (2019) [19] evaluated silver nanoparticles with aqueous extract of *Justicia spicigera* as an antimicrobial agent against foodborne bacteria (*Bacillus cereus*, *Klebsiella pneumoniae* and *Enterobacter aerogenes*) and phytopathogenic fungi (*Colletotrichum* sp., *Fusarium solani*, *Alternaria alternata* and *Macrophomina Phaseolina*) at a concentration of 100 mg/mL. They found effective antibacterial and antifungal activity against the organisms analyzed, especially against Bacillus cereus (10.0 ± 0.2 mm of inhibition) and *Macrophomina phaseolina* (80.95 ± 1.35 of Inhibition of radial growth). This lack of use represents a significant opportunity for research and the potential application of this plant in the food industry. Furthermore, the incorporation of *Justicia spicigera* into food matrices such as NB has been scarcely explored, which presents an opportunity to develop FFs using plant-based ingredients while taking advantage of the plant’s proven bioactive properties. The inclusion of such ingredients (i.e., plants or plant-derived components) with established bioactivity may enhance the cultural acceptance of the product, its nutritional impact, and its overall health benefits upon consumption.

Given the above, the present study aimed to develop and characterize (antioxidant capacity, water activity, color, texture profile, and proximate composition) a vegan nutritional bar as a functional food based on chickpeas and incorporating *Justicia spicigera*.

## 2. Materials and Methods

### 2.1. Preparing the Vegan Nutritional Bar (VNB)

The ingredients used in the preparation of the VNB included chickpeas (*Cicer arietinum*), whole wheat flour (*Triticum* spp.), pecan nuts (*Carya illinoinensis*), brown flaxseed *(Linum usitatissimum*), orange peel zest (*Citrus sinensis*), sodium bicarbonate (Promesa^®^), salt (La Fina^®^), vegetable oil (safflower oil, Oleico^®^), powdered soy milk (SoyaPac^®^), and monk fruit (*Siraitia grosvenorii*, NS^®^) as a sweetener. The chickpeas were cooked in water in a 1:4 (*w*/*v*) ratio in a pressure cooker for 45 min then ground into a puree used as the base for the bars. All ingredients were purchased from a local market in Montemorelos, Nuevo León, Mexico, and were selected for their freshness and high quality.

Fresh *Justicia spicigera* plants were obtained from a local market in Monterrey, Nuevo León, Mexico. The plant leaves were dried at room temperature (25 °C) on brown paper mats under aseptic conditions. They were then ground (80350G Hamilton Beach, Glen Allen, VA, USA) and incorporated at a concentration of 3.2% (*w*/*w*) (VNB_3.2) relative to the total weight of the formulation (Table 1). This percentage was selected based on preliminary internal studies, which demonstrated that it did not compromise the integrity of the dough used to form the bars. Moreover, increasing the amount of the plant led to a significantly darker color, increased bitterness, and a slightly crumbly texture. Furthermore, from an economic and industrial feasibility perspective, this concentration could facilitate integration into commercial-scale manufacturing processes.

The preparation of the VNB was carried out as follows: briefly, all dry ingredients (whole wheat flour, monk fruit, brown flaxseed, orange zest, baking soda, salt, and pecan nuts) were first mixed by hand. Subsequently, the cooked chickpeas and vegetable oil were incorporated. The ratio of liquid to solid ingredients used in the bar formulation was 56.1% liquid mixture and 43.9% solid ingredients. This process yielded a base formulation that was used to incorporate powdered *Justicia spicigera*. In the formulation, 3.2% (*w*/*w*) of *Justicia spicigera* was added. All ingredients were then thoroughly mixed and divided into 40 g portions. These were molded into bars measuring 9 cm in length and 3 cm in width and baked at 200 °C (Mabe, Louisville, KY, USA) for 18 min. The bars were allowed to cool to room temperature and subjected to visual inspection. Finally, the bars were immediately analyzed (i.e., proximate analysis, water activity, texture profile, color, and antioxidant capacity).

### 2.2. Proximate Analysis

The chemical composition of the VNB was conducted through a proximate analysis performed at the Department of Analytical Chemistry of the Universidad Autónoma de Nuevo León (UANL) and was determined according to the AOAC methods [20] and official Mexican norms. The analysis included the determination of the moisture content (AOAC 925.10, NOM-116-SSA1-1994), ash content (AOAC 940.26), fat content (NMX-F-615-NORMEX-2018), protein content (NMX-F-608-NORMEX-2011) using 6.25 as the nitrogen to protein conversion factor, crude fiber (AOAC 962.09), and nitrogen free extract (NFE) calculated by difference using Equation (1):

NFE (%) = 100 − (%*protein* + %*fat* + %*fiber* + %*ash* + %*moisture*) (1)

Each sample was measured in triplicate in independent samples.

### 2.3. Determination of Water Activity (a_w_)

Water activity was determined in individual samples using an AquaLab Series 3 TE device (Decagon Devices Inc., Pullman, WA, USA). The instrument was calibrated according to the manufacturer’s instructions, and each sample was measured in triplicate in independent samples.

### 2.4. Texture Profile Analysis

The texture profile of each sample was determined using a CT3 texture analyzer (Brookfield-Ametek, Middleborough, MA, USA) equipped with a flat cylindrical probe (TA7) and operated with the TexturePro CT V1.9 Build 35 software. A double compression test was performed using the flat probe. Since texture parameters can be highly variable due to sample heterogeneity and following the recommendations of the equipment used, each sample was analyzed in quintuplicate on independent samples. The parameters hardness, expressed in Newtons (N); deformation, expressed in millimeters (mm); adhesiveness, expressed in Joules (J) and fracturability, expressed in N, were determined.

### 2.5. Color Analysis

Color values (CIE L*, a*, and b*) of the outer part (center and ends) of the VNB samples were determined using a colorimeter (ColorFlex EZ, HunterLab, Reston, VA, USA). Measurements were performed in triplicate in independent samples. With this data the ΔE was calculated using Equation (2):

ΔE = √((L_2_ − L_1_)^2^ + (a_2_ − a_1_)^2^ + (b_2_ − b_1_)^2^) (2)

where L_1_, a_1_, and b_1_ are the CIELAB coordinates of VNB, and L_2_, a_2_, and b_2_ are the CIELAB coordinates of VNB_3.2.

The analyses of *a_w_*, texture, and color were conducted at the Department of Food Sciences, Faculty of Biological Sciences, UANL.

### 2.6. Determination of Percentage of Radical Scavenging Activity

The percentage of radical scavenging activity (antioxidant capacity) of the VNB samples was determined using the DPPH (2,2-Diphenyl-1-picrylhydrazy) (Merck^®^) method [21]. Briefly, the VNB was ground and extracted with methanol (Tedia^®^) at a 1:4 (*w*/*v*) ratio. A solution of the methanolic extract was then prepared at a concentration of 100 μg/mL. Subsequently, 1 mL of this solution was mixed with 1 mL of a DPPH solution (80 μg/mL) in methanol. The mixtures were kept in the dark at room temperature for 30 min. Absorbance was measured at 517 nm using a spectrophotometer (Thermo Fisher Scientific Genesys 20, Waltham, MA, USA).

On the other hand, the ABTS (2,2′-azino-bis(3-ethylbenzothiazoline-6-sulfonic acid) (Merck^®^) method is based on the reduction in the green ABTS radical cation. A volume of 25 μL of each solution (VNB or VNB_3.2) was added to 1 mL of ABTS methanol solution (7 mM). After 7 min, absorbances were measured at 734 nm (Thermo Fisher Scientific Genesys 20, Waltham, MA, USA).

The percentage of free radical inhibition (%I) was calculated using Equation (3):

%I = ((ABS_0_ − ABS_sample_)/ABS_0_) × 100 (3)

where ABS_0_ is the absorbance of the radical (DPPH or ABTS) in methanol, and ABS_sample_ is the absorbance of the sample with the radical. The samples were analyzed in triplicate in independent samples.

### 2.7. Statistical Analysis

Descriptive data are reported as mean and standard deviation (SD). Statistical analysis was performed on independent batches. Data were checked for normality using Shapiro–Wilk tests. For normally distributed data (*p* > 0.05), independent samples ANOVA were used, and for non-normally distributed data, Mann–Whitney U tests were used (*p* < 0.05) using RStudio^®^ software version 2025.09.2+418.

## 3. Results and Discussion

### 3.1. Formulation of the Vegan Nutritional Bar (VNB)

**Figure 1:**
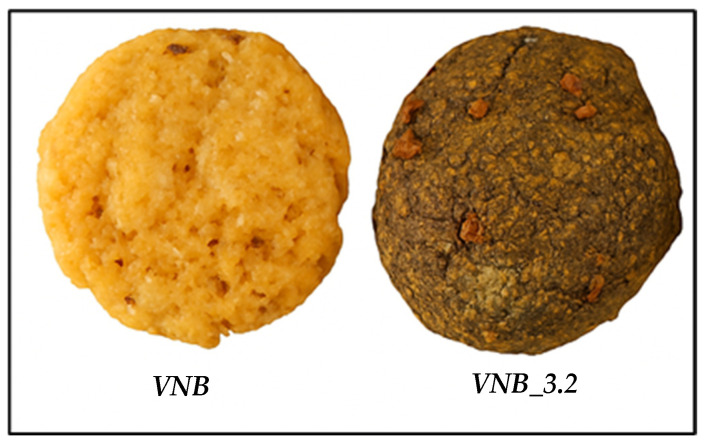
Vegan nutritional bar dough. VNB, without *Justicia spicigera* powder, and VNB_3.2, with 3.2% *Justicia spicigera*.

The development of the VNB focused on creating a balanced and functional food product using plant-based ingredients with high nutritional value (Figure 1).

VNBs are widely recognized as effective delivery systems for functional ingredients due to their stability, portability, and consumer acceptance across all age groups and dietary preferences [22,23]. In recent years, several studies have demonstrated the feasibility of incorporating legumes, seeds, and plant extracts into bar formulations to improve nutritional quality and bioactivity while maintaining acceptable color properties as reported by Atik et al. [24] and Felisiak et al. [10]. For this study, chickpeas (*Cicer arietinum*) were selected as the main protein source due to their excellent nutritional profile, including high protein content [25,26]. The protein quality of chickpeas is considered better than that of other legumes, as they contain 8 of the 9 essential amino acids [27]. Chickpeas are considered an affordable source of protein compared to animal-based options. This is particularly relevant for individuals with limited income, who may face challenges in accessing nutritionally balanced foods. Chickpeas are a good source of carbohydrates and also have a higher content of fat, ash, and fiber. Additional ingredients such as pecan nuts, brown flaxseed, and orange zest contributed to essential fatty acids, micronutrients, and flavor-enhancing properties. The initial stage of the formulation (i.e., without *Justicia spicigera*) was optimized through internal pilot tests that mainly analyzed the texture and appearance at the discretion of the researchers. During the incorporation of the plant into the initial formulation, it was observed that the dough with higher percentages of *Justicia spicigera* (>3.2%) had difficulty maintaining its structural integrity, a much darker color, and a brittle texture that hindered its formation. The addition of *Justicia spicigera* powder was intended to improve the functional properties of the bars thanks to its previously reported antioxidant, anti-inflammatory and hepatoprotective effects.

### 3.2. Proximate Analysis of VNB and VNB_3.2

Proximate analysis provides an initial assessment of the nutritional quality of food products, offering essential information for the design of FFs. Each analyzed parameter plays a specific nutritional and technological role. In the case of the developed nutritional bar, plant-based ingredients were selected for their richness in proteins, polyunsaturated fats, and natural sweeteners, with the aim of achieving adequate nutritional quality. The results of the proximate analysis for both the control bar (VNB) and the bar enriched with 3.2% *Justicia spicigera* (VNB_3.2) are presented in Table 2.

**Moisture** content directly influences the product’s shelf life and microbiological stability. In this study, the formulations showed values of 14.74 ± 0.29% (VNB) and 12.32 ± 0.17% (VNB_3.2), respectively. The observed reduction in moisture content in the VNB_3.2 formulation (12.32 ± 0.17%) compared to the control (14.74 ± 0.29%) may be attributed to the high phenolic and flavonoid content, such as kaempferol and peonidin derivatives, of *Justicia spicigera* powder. These compounds are known to bind water molecules, reducing the availability of free moisture in the matrix [28]. These values are similar to those reported by Alfheeaid et al. (2023) [23], who obtained moisture values of 11.22 ± 0.08 and 13.24 ± 0.04% in fruit bars. Also, Dar et al. [29] evaluated functional bars enriched with black chickpeas, pumpkin seeds, and coconut, reporting moisture levels around 16%. Such values are considered acceptable for maintaining product stability during storage.

**Ash** content reflects the total mineral content, which is important for various physiological functions. The VNB formulation exhibited 2.0 ± 0.025%, while VNB_3.2 presented a slight increase to 2.37 ± 0.081%, likely due to the mineral contribution of *Justicia spicigera* (i.e., potassium, calcium acetate and oxalate, sulfate and sodium chloride) present in the plant [30]. These values align with those found by Bourekoua et al. [31], who reported ash content between 1.8% and 2.0% in energy and fiber-rich bars processed under different treatments.

**Fat** content in the formulations can be attributed to ingredients such as pecans, brown flaxseed, and vegetable oil, with VNB showing 23.87 ± 0.06% and VNB_3.2 reaching 26.39 ± 0.23%. Polyphenols and flavonoids present in *Justicia spicigera* may interact with lipids during baking, reducing lipid oxidation and promoting greater retention of fats in the final product [32]. These values are higher than those reported by AlJaloudi et al. [33] in bars made with seeds and dried fruits, which ranged from 8% to 18%. However, in formulations containing oilseeds and nuts, such values are expected and desirable, as they provide essential fatty acids, enhance flavor, and improve texture.

**Protein** content increased significantly from 6.75 ± 0.07% in the VNB formulation to 14.31 ± 0.01% in VNB_3.2, highlighting the nutritional enhancement achieved by incorporating *Justicia spicigera* powder. Although *Justicia spicigera* is primarily known for its antioxidant and pigmenting properties, recent studies have identified the presence of glycosylated flavonoids such as kaempferitrin, as well as free amino acids and nitrogen-containing compounds which contribute to total nitrogen measured via the Kjeldahl method [28,34,35]. The absolute protein content of *Justicia spicigera* is not high compared to legumes such as chickpea, its inclusion in the bar may enhance protein quality through the presence of functional amino acids and synergistic interactions with other ingredients. Medicinal plants like *Justicia spicigera* have been shown to contain free amino acids and peptides that further contribute to the nutritional profile of functional foods [36]. Jabeen et al. [37], reported higher protein content (22.5%) in bars enriched with chickpeas and rice due to dual cereal enrichment, the value of 14.3% in the present study falls within the 13–17% range typically observed in legume-based bars, as reported by Kumar et al. [38].

**Crude fiber** content decreased from 6.42 ± 0.08% (VNB) to 3.51 ± 0.09% (VNB_3.2), which may be attributed to the relatively low fiber contribution of *Justicia spicigera* powder. Although this plant is rich in phenolic compounds, it is not a significant source of dietary fiber. Furthermore, the incorporation of *Justicia spicigera* powder may have displaced fiber-rich ingredients such as whole wheat flour and chickpeas, resulting in a dilution effect. It is also important to note that crude fiber analysis may underestimate total dietary fiber, especially in formulations containing soluble fibers and non-cellulosic polysaccharides [39]. A similar trend was reported by Jabeen et al. [37], who observed a decrease in crude fiber content from 7.16% to 5.81% in protein-rich energy bars, which they attributed to the inclusion of apricot paste in their formulations.

**NFE** represents the fraction of soluble carbohydrates and other easily digestible non-nitrogenous compounds, serving as an important indicator of the product’s energy value. The VNB formulation showed an NFE content of 46.75 ± 0.11%, while VNB_3.2 exhibited a slightly lower value of 41.08 ± 0.21%. The presence of polyphenols and non-digestible components in *Justicia spicigera* may interfere reducing the amount of NFE [40]. These results are consistent with those reported by Costa Maia et al. [41], who found available carbohydrate contents ranging from 38.5% to 44.7% in bars made with various legume flours (e.g., pigeon pea, cowpea, and guandu). This suggests that chickpea-based formulations maintain an energy profile comparable to that of other commonly used legumes in functional snack bar formulations. From a nutritional standpoint, this can be advantageous, as it may promote a lower glycemic index and enhance functional potential. Furthermore, it is important to note that VNB does not contain added sugars; therefore, the carbohydrates present are all of natural origin.

### 3.3. Determination of a_w_ of VNB and VNB_3.2

*a_w_* is a critical parameter that influences the microbiological stability, shelf life, and overall quality of food products. It reflects the amount of free water available for microbial growth and chemical reactions, beyond the total moisture content [37]. In this study, the *a_w_* values of the formulations were 0.6915 ± 0.0008 for VNB and 0.7553 ± 0.0006 for VNB_3.2, values with significantly different (*p* < 0.05) (Table 3). Structural changes in the matrix due to the incorporation of plant powder may also influence water mobility and retention. Jabeen et al. [42], state that foods with an *a_w_* between 0.6 and 0.9 are sufficiently protected against microbial spoilage and can maintain stability. Therefore, bars within this range can be expected to have an extended shelf life. Coello et al. [43], mention that obtaining nutritional bars with a water activity lower than 0.78 is an important factor since it leads to prolonging the shelf life of these foods. Low *a_w_* values are often associated with improved oxidative stability, particularly in formulations with high fat content [24]. The water activity observed in the VNB_3.2 formulation, being an intrinsic factor of the food, suggests a longer shelf life and improved microbiological stability. Therefore, future studies should explore additional preservation strategies such as modified atmosphere packaging and edible coatings.

The water activity observed in the VNB_3.2 formulation suggests a shelf life and enhanced microbiological stability, reinforcing its potential as a plant-based functional snack bar.

### 3.4. Texture Profile Analysis of VNB and VNB_3.2

Textural characterization (e.g., hardness, deformation, adhesiveness, and fracturability) is important for the acceptance of nutritional bars, since parameters such as hardness and fracturability determine the consumer experience and overall acceptability of the product. The results of the present study are shown in Table 3. The adhesiveness of both formulations (VNB and VNB_3.2) was 0 J, indicating that no measurable stickiness was observed under the test conditions, therefore the result is omitted in Table 3.

The results obtained in the texture analysis (Table 3) showed no statistically significant differences between samples VNB and VNB_3.2. Hardness and deformation remained very similar between the two formulations, while fracturability increased in the VNB_3.2 sample, albeit with higher variability. This may be positive since the incorporation of *Justicia spicigera* powder does not interfere with the textural properties of VNB. Ho et al. [44], reported hardness between ~6 and ~8 N for green banana flour bars, showing that our formulations are in this range. Such moderate hardness may be advantageous, as it enhances chewability and textural acceptability.

Deformation values were virtually identical between samples (≈4.0 mm), indicating that the inclusion of *Justicia spicigera* did not significantly affect the bars’ elasticity.

Fracturability, defined as the force at the first crisp break, was slightly higher in VNB_3.2 (7.06 ± 3.59 N vs. 5.71 ± 4.79 N). This suggests a marginally firmer structure, potentially due to the reinforcing particulates from *Justicia spicigera* powder. Comparable behavior was observed in cereal bars where binding agents increased firmness and fracture resistance [45].

### 3.5. Color Analysis of VNB and VNB_3.2

Color is a critical quality attribute in food products, influencing consumer perception and acceptability. The color parameters L* (lightness), a* (red-green axis), and b* (yellow-blue axis) were measured to evaluate the visual differences between the control formulation (VNB) and the formulation with 3.2% *Justicia spicigera* powder (VNB_3.2). The results are presented in Table 4.

**Figure 2:**
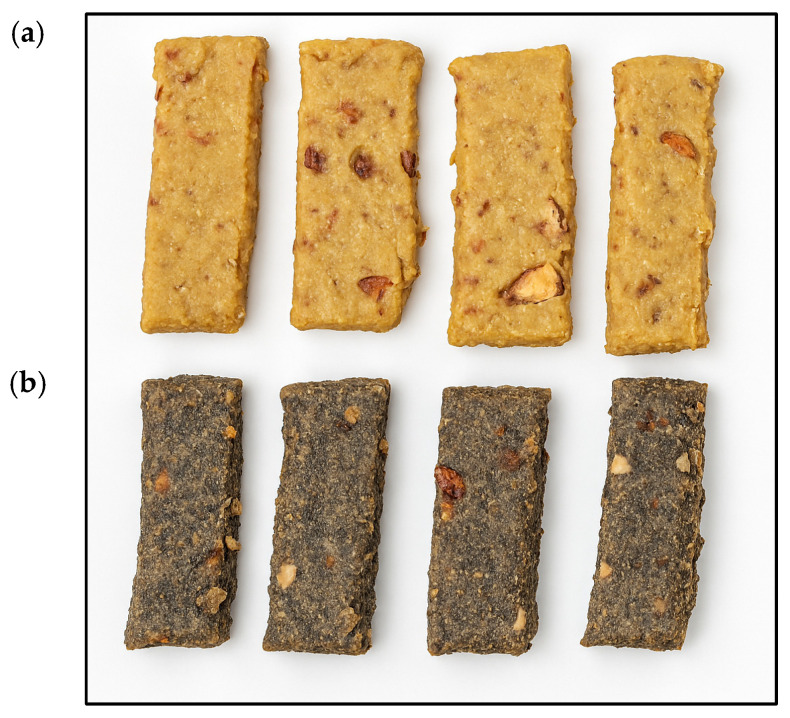
(**a**) vegan nutritional bar (VNB) without *Justicia spicigera* powder, and (**b**) bar with 3.2% incorporated *Justicia spicigera* (VNB_3.2).

The color analysis revealed statistically significant differences between the VNB and VNB_3.2 samples across all parameters (L*, a*, b*), with a total color difference of ΔE = 18.78, indicating a highly perceptible visual change. Specifically, VNB exhibited higher luminosity (L* = 36.28 ± 0.00), greater redness (a* = 12.55 ± 0.01), and stronger yellowness (b* = 26.66 ± 0.02) compared to VNB_3.2 (L* = 27.79 ± 0.04, a* = 4.04 ± 0.01, b* = 12.23 ± 0.04). These results coincide with those seen in Figure 2.

The sample without *Justicia spicigera* (VNB) exhibited a significantly higher L* value, indicating a lighter appearance, which is consistent with the natural color of chickpea on based formulations. In contrast, the incorporation of *Justicia spicigera* powder markedly reduced the L* value, resulting in a darker product. This shift can be attributed to the presence of anthocyanins and other phenolic compounds in *Justicia spicigera*, known to impart deep purple or green hues depending on their chemical environment, in addition to the dark color of the plant’s powder itself [30,46].

The a* value decreased from 12.55 ± 0.01 in VNB to 4.04 ± 0.01 in VNB_3.2. This may reflect the replacement of ingredients with reddish-brown hues (such as pecan and chickpea) with the dark-colored *Justicia spicigera* powder. Similarly, b* values dropped from 26.66 ± 0.02 to 12.23 ± 0.04, reflecting a significant decrease in the yellow component, further supporting the influence of phenolic pigments.

These findings are consistent with recent studies on nutritional bars. Fanari et al. [47] investigated the incorporation of microalgae (*Spirulina* and *Chlorella*) into energy bars and found that even low inclusion levels significantly altered color, particularly reducing L* and increasing greenish or brownish tones depending on the strain. These changes were linked to pigment degradation and oxidative reactions during mixing and baking.

From a product development perspective, although the addition of *Justicia spicigera* modified the visual profile of the bar, the darker color may serve as a cue for the presence of functional ingredients, which can enhance consumer perception of health benefits. Nevertheless, future sensory studies should be conducted to assess the visual acceptability among target populations.

### 3.6. Percentage of Radical Scavenging Activity of Vegan Nutrition Bar

The antioxidant activity of the vegan nutritional bar was assessed using DPPH and ABTS assays, widely recognized for their reliability and sensitivity in assessing free radical scavenging capacity. These methods measure the ability of antioxidants to neutralize radicals, providing insight into the product’s functional potential and thus its ability to prevent oxidative stress associated with chronic diseases [48,49]. In this study, antioxidant activity was expressed as a percentage of radical inhibition, revealing statistically significant differences between treatments (*p* < 0.05) (Table 5).

The formulation enriched with *Justicia spicigera* (VNB_3.2%) presented 62.6% more antioxidant capacity than VNB in the DPPH assay and 9.7% more antioxidant capacity than VNB in the ABTS assay. These findings suggest that the inclusion of *Justicia spicigera* powder improves the radical scavenging capacity of the product, probably due to its high content of flavonoids, anthocyanins and other bioactive phenolic compounds (i.e., kaempferitrin, hesperidin, kaempferol, naringenin, among others) [30]. The health benefits of antioxidants are now widely recognized, linking them to the prevention of numerous diseases, such as heart disease and cancer [50,51].

This result is consistent with previous studies that have demonstrated the presence of flavonoids, anthocyanins, and other phenolic compounds with strong electron-donating capacity in *Justicia spicigera*. Additionally, Gumul et al. [52], reported that the inclusion of plant-based ingredients rich in phenolic compounds in functional bars contributes to enhanced antioxidant activity and a potential extension of shelf life by inhibiting lipid oxidation. This behavior was also reported by Eid et al. [53], who found an antioxidant activity ranging from 60 to 75% in bars fortified with different levels of *Moringa oleifera* leaves powder. Moreover, due to the high content of unsaturated fats in the formulation (pecans, brown flaxseed, and vegetable oil), enhanced antioxidant activity not only improves the potential health benefits of the product but may also contribute to greater oxidative stability during storage, helping to prevent lipid oxidation and rancidity.

The above results demonstrated that the vegan nutritional bar with *Justicia spicigera* (VNB_3.2) powder may be a promising functional food alternative for populations at risk of malnutrition.

## 4. Conclusions

The vegan nutritional bar with *Justicia spicigera* showed higher antioxidant capacity, protein content, and fat content. The two bars, with and without *Justicia spicigera*, presented a similar texture profile. This study highlights the potential of developing plant-based nutritional bars as functional food solutions to address nutritional deficiencies and promote health. The chickpea-based formulation enriched with *Justicia spicigera* represents a promising strategy to combine the nutritional quality of plant products with natural bioactive compounds. Chickpeas provide a balanced source of vegetable protein and fiber, while *Justicia spicigera*, a traditionally used medicinal plant, offers antioxidant and health-protective properties that enhance the bar’s functional value. Some limitations are acknowledged, such as the lack of a formal sensory evaluation with a standardized panel. Future studies are required to evaluate other biological activities provided by the presence of *Justicia spicigera*, as well as the potential reformulation of ingredients to improve consumer acceptability.

Finally, the integration of these ingredients resulted in a product with favorable physical characteristics and nutritional potential, making it suitable for populations seeking sustainable, plant-based alternatives and culturally acceptable alternatives. Furthermore, with the growth in the nutrition bar market, this product can be easily scaled up, as all ingredients are locally sourced and readily available. This innovation contributes to the growing field of FFs development and could serve as a foundation for future research focused on clinical efficacy and shelf life stability under real-life conditions.

## Figures and Tables

**Table 1:** Ingredients of the vegan nutritional bar (VNB) and bar with incorporated *Justicia spicigera* (VNB_3.2).

Parameter	Experimental Bars	
	VNB ^A^	VNB_3.2 ^B^
Chickpea (g)	29	29
Whole wheat flour (g)	28	28
Pecan nuts (g)	13.50	13.50
brown flaxseed (g)	0.60	0.60
Orange Zest (g)	1.70	1.70
Vegetable oil (g)	15.30	15.30
Powdered soy milk (g)	4	4
Monk Fruit (g)	7	7
*Justicia spicigera* powder (%)	---	3.2

^A^ vegan nutrition bar without *Justicia spicigera*; ^B^ vegan nutrition bar with 3.2% of *Justicia spicigera*.

**Table 2:** Proximate analysis evaluation results of vegan nutritional bar (VNB) and bar with incorporated *Justicia spicigera* (VNB_3.2).

Parameter (%)	VNB ^A^	VNB_3.2 ^B^
Moisture	14.74 ± 0.29 ^a^	12.32 ± 0.17 ^b^
Ash	2.00 ± 0.025 ^a^	2.37 ± 0.081 ^b^
Fat	23.87 ± 0.06 ^a^	26.39 ± 0.23 ^b^
Protein	6.75 ± 0.07 ^a^	14.31 ± 0.01 ^b^
Crude fiber	6.42 ± 0.08 ^a^	3.51 ± 0.09 ^b^
NFE	46.75 ± 0.11 ^a^	41.08 ± 0.21 ^b^

^A^ vegan nutrition bar; ^B^ vegan nutrition bar with 3.2% *Justicia spicigera*; letters a and b indicate statistically different groups based on post hoc testing. Values with significantly different (*p* < 0.05) test ANOVA o Mann–Whitney U based on normality. (*n* = 3; $\bar{x}$ ± SD).

**Table 3:** Average values of the texture parameters and *a_w_* of vegan nutritional bar (VNB) and bar with incorporated *Justicia spicigera* (VNB_3.2).

Parameter	VNB ^A^	VNB_3.2 ^B^
*a_w_*	0.6915 ± 0.0008 ^a^	0.7553 ± 0.0006 ^b^
Hardness (N)	8.58 ± 1.92 ^a^	8.44 ± 0.89 ^a^
Deformation (mm)	4.00 ± 0.009 ^a^	4.00 ± 0.004 ^b^
Fracturability (N)	5.71 ± 4.79 ^a^	7.06 ± 3.59 ^b^

^A^ vegan nutrition bar; ^B^ vegan nutrition bar with 3.2% *Justicia spicigera*; letters a and b indicate statistically different groups based on post hoc testing. Values with significantly different (*p* < 0.05) test ANOVA o Mann–Whitney U based on normality. (*n* = 5; $\bar{x}$ ± SD).

**Table 4:** Average values of lightness (L*), intensity of red color (a*), intensity of yellow color (b*) of vegan nutritional bar (VNB) and bar with incorporated *Justicia spicigera* (VNB_3.2).

Sample	L* (Lightness)	a* (Red-Green)	b* (Yellow-Blue)
VNB ^A^	36.28 ± 0.00 ^a^	12.55 ± 0.01 ^a^	26.66 ± 0.02 ^a^
VNB_3.2 ^B^	27.79 ± 0.04 ^b^	4.04 ± 0.01 ^b^	12.23 ± 0.04 ^b^
	ΔE = 18.87		

^A^ vegan nutrition bar; ^B^ vegan nutrition bar with 3.2% *Justicia spicigera*; letters a and b indicate statistically different groups based on post hoc testing. Values with significantly different (*p* < 0.05) test ANOVA o Mann–Whitney U based on normality. (*n* = 3; $\bar{x}$ ± SD).

**Table 5:** Antioxidant activity of vegan nutritional bar (VNB) and bar with incorporated *Justicia spicigera* (VNB_3.2).

Sample	Percentage Inhibition	
	DPPH (%)	ABTS (%)
VNB ^A^	47.61 ± 1.13 ^a^	70.90 ± 3.10 ^a^
VNB_3.2 ^B^	77.46 ± 5.37 ^b^	84.85 ± 0.81 ^b^

^A^ vegan nutrition bar; ^B^ vegan nutrition bar with 3.2% *Justicia spicigera*; letters a and b indicate statistically different groups based on post hoc testing. Values with significantly different (*p* < 0.05) test ANOVA o Mann–Whitney U based on normality. (*n* = 3; $\bar{x}$ ± SD).

## Data Availability

The original contributions presented in the study are included in the article, further inquiries can be directed to the corresponding author.
